# The Use of Stem Cell Differentiation Stage Factors (SCDSFs) Taken from Zebrafish Embryos during Organogenesis and Their Role in Regulating the Gene Expression of Normal and Pathological (Stem) Cells

**DOI:** 10.3390/ijms21144914

**Published:** 2020-07-12

**Authors:** Pier Mario Biava

**Affiliations:** Scientific Institute of Research and Care Multimedica, Via Milanese 300, 20099 Sesto S. Giovanni (Milano), Italy; piermario.biava@gmail.com

**Keywords:** embryo, stem cell, gene regulation, cancer cell reprogramming, psoriasis, senescence prevention, neurodegeneration, tissue regeneration

## Abstract

Studies conducted on Zebrafish embryos in our laboratory have allowed for the identification of precise moments of organogenesis in which a lot of genes are switched on and off, a sign that the genome is undergoing substantial changes in gene expression. Stem cell growth and differentiation stage-factors present in different moments of organogenesis have proven to have different specific functions in gene regulation. The substances present in the first stages of cell differentiation in Zebrafish embryos have demonstrated an ability to counteract the senescence of stem cells, reducing the expression of the beta-galactosidase marker, enhancing the genes *Oct-4*, *Sox-2*, *c-Myc*, *TERT*, and the transcription of Bmi-1, which act as key telomerase-independent repressors of cell aging. The molecules present in the intermediate to late stages of cell differentiation have proven to be able to reprogram pathological human cells, such as cancer cells and those of the basal layer of the epidermis in psoriasis, which present a higher multiplication rate than normal cells. The factors present in all the stages of cell differentiation are able to counteract neurodegeneration, and to regenerate tissues: It has been possible to regenerate hair follicles in many patients with androgenetic alopecia through transdermal administration of stem cell differentiation stage factors (SCDSFs) by means of cryopass-laser.

## 1. Introduction

**Figure 1:**
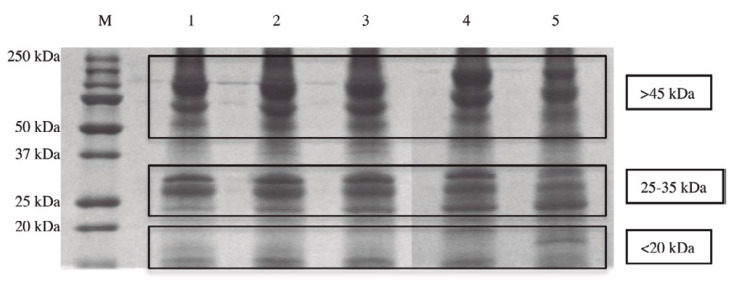
The different composition of proteins taken in the five stages of cell differentiation analyzed on a one-dimensional sodium dodecyl sulphate-polyacrylamide gel electrophoresis (SDS-PAGE).

Many data presented in scientific publications report that the administration of carcinogenic substances during embryo organogenesis does not cause tumors in offspring [1,2], while it is possible to induce tumors in offspring after the end of the cell differentiation process [3,4,5,6]. Such data seem to indicate that cancer can be considered a reversible process, which can be controlled by the factors present during organogenesis. Taking that into account, it was decided that a systematic study of the cellular differentiation process should be conducted. Many experiments were carried out in our laboratories using the Zebrafish embryo as a model to study the cell differentiation process for the following reasons: first, that it is easy to establish the exact time of zebrafish egg fertilization. This is important for standardizing all the experiments, with the goal of obtaining repeatable results over time in different research studies; second, notwithstanding their size and relative simplicity, the proteins extracted from Zebrafish might have been overall evolutionarily conserved in humans. In fact, all the different protein types taken from Zebrafish embryos after the beginning of stem cell differentiation were identified in our research using liquid chromatography mass spectrometry testing [7]. It was demonstrated that the identified proteins, which represented 98% of the molecules isolated from the different stages of cell differentiation, are important for the metabolism of mitochondria, for their function in anti-oxidant defense, for immune response, and for the whole metabolism of the cells (the remaining 2% of the molecules isolated were nucleic acids). Moreover, the research showed that the proteins taken from Zebrafish embryos have been evolutionarily conserved in human species, as expected. The different molecular weight of the proteins present in the five stages of organogenesis (50% of epiboly, tail bud, 5 somites, 20 somites, and the beginning of pharyngula) were identified using the PolyAcrylamide Gel Electrophoresis (SDS-PAGE) method. In all five stages of cell differentiation, three main protein clusters are distinguishable on the basis of their molecular weight (Figure 1).

These substances represent the system which is able to regulate the gene expression of all the genes of all the cells of the body. Indeed, all the substances which are able to determine which genes will remain active and which will not; which proteins will be synthesized and which will not; which molecular communication mechanisms will remain operational and which will not; and in what way codifying genes will interact with their products are present at that time. These substances, which together make up the program that gives rise to a new life, act as regulators of gene expression so that when the differentiation process has been completed, all the differentiated cells have the same basic DNA, but the part of active codifying genes in each differently specialized cell is specifically different and represents only a fraction of the entire DNA. When the process of cell differentiation is over and a complete organism has been formed, we cannot study all the functions of the gene regulation system because the different components of this system are subdivided into various tissues and organs and are present in an organ only as the part that controls the gene expression of the cells of that particular organ. Therefore, in an adult organism, we cannot study all the different functions of the gene regulation system, but it is possible to provide specific information regarding regulating the genes of normal and of cancer cells. The study of the functions of the gene regulation system in Zebrafish embryos has demonstrated that they differ a lot according to the different stages of cell differentiation. The substances present in the moments before or just at the beginning of stem cell differentiation are significant in activating important genes responsible for counteracting human cell senescence. In fact, it was demonstrated that the factors taken from the Zebrafish embryo just before the middle blastula–gastrula are able to activate all the genes which counteract cell senescence. [7,8,9,10]. Similarly, the substances present during the stages in which cell differentiation events take place are able not only to differentiate normal stem cells but also to reprogram cancer cells to a normal phenotype [11].

The aim of this paper is to report on how the different substances which are present in the different specific moments of organogenesis are able to control the gene expression of the cells of an organism very specifically, in order to obtain very specific results in the fields of tissue regeneration and of cell reprogramming. The substances present in specific stages of cell differentiation make up specific, complex programs, which act as a whole, considering that each new step of stem cell differentiation is only possible when specific instructions on gene regulation are entirely present. Each step of cell differentiation represents a dramatic change in gene expression, because at each step, hundreds of genes are simultaneously regulated by the complex networks of substances present in each stage, and no differentiation steps or important changes in gene expression are possible if single molecules are used. Thus, in order to obtain significant results in regenerating tissues or in reprogramming pathological stem cells, such as cancer cells, in which many mutations or many alterations of genes expression are usually present, we have to use specific programs of cell differentiation because single molecules cannot obtain any significant results.

## 2. The Reprogramming Treatments of Cancer Stem-Like Cells: The Results of the Experiments In Vitro and In Vivo

Different human tumor lines (glioblastoma multiforme, hepatocellular carcinoma, melanoma, breast cancer, kidney adenocarcinoma, and acute lymphoblastic leukemia) have been treated with factors taken from Zebrafish embryos in different developmental stages: (a) morula stage, in which totipotent stem cells are present, predominantly; (b) 50% of epiboly, in which the process of cell differentiation begins; (c) five somite stage; and (d) twenty somite stage, in which stem cells in the intermediate to final stages of cell differentiation are present.

All cell lines have shown a significant slowdown in their growth only when treated with factors taken during the cell-differentiation stages, while weak tumor growth was obtained with factors taken in the phase of stem cell multiplication [12].

The effects of stem cell differentiation stage factors (SCDSFs) on tumor growth were also observed in vivo after subcutaneous injection of primary Lewis lung carcinoma cells into C57BL/6 female syngeneic mice weighing 18–20 g. A highly significant difference was noted (*p* < 0.001) between treated and control mice in favor of the treated mice [7,13]. All these experiments confirm the hypothesis that only when the factors taken during the stages of differentiation are used, is it possible to address tumor cells, pushing them toward the normal path of differentiation. These factors appear in the phases of cell differentiation, and they are absent in the stages of mere multiplication.

A lot of research was carried out in order to understand which molecular events are involved in the tumor-growth inhibition mechanism. It was demonstrated that molecules such as p53 and pRb, which are important in the control of the cell cycle, are involved in it. Indeed, a p53 transcriptional regulation was obtained, highlighted by a considerable increase of the p53 protein’s concentration in the cells of some tumor lines, such as glioblastoma and melanoma. This has been evaluated by means of a cytofluorimetric analysis, as well as the immune-histochemical method, after treatment with cell-differentiation factors [14].

The slowdown of tumor growth on other tumor lines, such as kidney adenocarcinoma, is due to the post-translational regulation of the retinoblastoma protein (pRb) with a change in the relation between the protein’s phosphorylated and non-phosphorylated form [15], resulting in a prevalence of the non-phosphorylated shape. The non-phosphorylated form stops the cell cycle of cancer cells, preventing the transcription of the *E2F-1* gene.

Lastly, programmed cell-death events (apoptosis) were studied. The analysis was carried out on colon adenocarcinoma cells, demonstrating the activation of an apoptotic pathway as well as a cell-differentiation pathway. Within a culture of colon tumor cells, there was a significant increase of cells in apoptosis, as well as cells with a high concentration of cell-differentiation markers [16]. Therefore, the molecular mechanisms at the basis of the slowdown of tumor growth due to treatment with SCDSF can be summarized as follows: a stop to the cell cycle and genetic damage repair, which leads to cell re-differentiation, or cell death of the tumor cells (if repair is no longer possible).

Just recently, researchers have observed that some factors from Zebrafish embryos during a specific developmental phase can inhibit breast cancer cell multiplication and its migration capabilities. In particular, it has been observed that SCDSFs significantly counteract the proliferation of breast cancer cells because they not only reduce cell multiplication and enhance apoptosis but also dramatically inhibit tumor spreading and metastasis. Moreover, it has been demonstrated that SCDSFs are also able to inhibit migration and invasiveness of the breast cancer cells in the epithelial–mesenchymal transition phase after TGF-β1 stimulation. This happens because of a modulation of the E-cadherin/β-catenin pathway and a dramatic reduction in vinculin, as well as downregulation of TCTP and a concomitant increase in p53 levels [17].

Furthermore, in order to ascertain if these embryonic factors could interact with chemotherapeutical substances, some experiments using 5-Fluorouracil (5-Fu), both alone and in association with SCDSFs, were carried out. It was demonstrated that in human colon cancer (Caco2) treated with Zebrafish stem cell differentiation stage factors in association or not with 5-Fu in the sub-pharmacological therapeutic range, cell proliferation was significantly reduced by SCDSF alone, while SCDSF+5-Fu led to a very dramatic growth-inhibition. These results were obtained using the following methods: whole cell-count, flow-cytometry analysis, and study of apoptotic parameters [18]. These data suggest that SCDSFs significantly improve chemotherapy efficacy.

In addition, other recent studies have demonstrated that the factors which are present in mesenchymal stem cells (MCS) transferred by exosomes can inhibit tumor growth because they contain substances which can lead tumor cells toward differentiation or apoptosis.

Stem cells are found and can be isolated in multiple tissues of our bodies, such as adipose tissue, bone marrow, and the umbilical cord. Some functions of these cells are to replace damaged cells and to cause differentiation.

The research on umbilical cord stem cells derived from Wharton’s jelly is very interesting. Indeed, umbilical cord stem cells derived from Wharton’s jelly possess a unique transcriptome that shows pro-apoptotic and anti-cancer properties [19]. A study conducted in 2012 demonstrated that human Wharton’s jelly stem cell extracts significantly attenuated tumor growth in different types of cancer cells in vitro [20], and in another study, it was observed that exosomes from umbilical-cord-derived MCS inhibited the growth of breast cancer cells in both in vitro and in vivo studies [21].

Moreover, a report published in 2015 detailed the therapeutic potential that miR-134 derived from exosomes of patients with triple-negative breast cancer have as a tumor suppressor and confirmed that miR-134 reduced triple-negative breast cancer aggression and increased drug sensitivity [22]. It was also demonstrated that exosomes secreted by mesenchymal stem cells (MSC) have the ability to control tumor growth, due to the specific substances which they transfer. Exosomes derived from MSC contain multiple cargoes and proteins that may control different relevant metabolic pathways of malignant cells [23].

In 2012, researchers evaluated if exosomes from MSC of human bone marrow can inhibit in vivo and in vitro growth of multiple tumors. The study revealed that exosomes secreted from MSC of human bone marrow inhibitet tumor progression of various types of cancer cells, such as hepatoma, ovarian tumors, and Kaposi’s sarcoma [24]. Many other studies about cancer cell reprogramming were conducted by different authors, who obtained the same results, which demonstrated that it is possible to revert cancer cells to a normal phenotype using stem-cell factors derived from the embryonic microenvironment [25,26,27,28,29,30,31,32].

## 3. SCDSFs: Results from Clinical Trials on Intermediate-Advanced Hepatocellular Carcinoma (HCC) and on Colon Cancer

A randomized, controlled trial [33] was conducted from January 2001 to the end of April 2004 on 179 patients affected by HCC in an intermediate-advanced stage for which no treatment of consolidated efficacy was possible, such as liver resection or transplantation, chemoembolization, ablation with radiofrequency, or other types of treatment. On the basis of the previous research described above, it was possible to conceive and develop a nutraceutical product containing the proteins as previously recorded, collected in the stages of cell differentiation in which they had demonstrated well-documented anti-cancer properties. Considering the low molecular weight of these proteins, sublingual absorption was proposed. The results showed a statistically significant difference in favor of the group of treated patients, in comparison with the control group. There was a 19.8% rate of regression, and a 16% rate of stabilization, with an overall survival rate after forty months of more than 60% of the patients who responded, compared to 10% of the non-responding patients. Great improvement of performance status was registered in a great majority of patients (82.6%) [33].

A more recent clinical trial conducted by the Scientific Institute of Research and Care Humanitas of Milan on patients with hepatocellular carcinoma in advanced stage has confirmed the role of SCDSFs in producing a complete response [34]. Lastly, a recent clinical trial conducted by the Institute of Oncology of University La Sapienza of Rome, comparing a group of patients with advanced-stage colon cancer treated with Regorafenib alone with another group treated with Regorafenib plus SCDSFs, demonstrated a statistically significant increase in survival in the latter group. Similarly, a significant difference was observed regarding the performance status, which was preserved in patients treated with SCDSFs and Regorafenib, in comparison with the patients treated with Regorafenib alone [35].

Other papers about the efficacy of SCDSFs in cancer treatments were published by different authors, who suggested the use of SCDSFs as integrative treatment to the traditional therapies of consolidated efficacy [36,37,38]. It is also worth noting that a declaration of a committee of oncologists published in a recent book suggests using SCDSFs as integrative treatments in oncology [39].

Many other experiments on SCDSFs have been undertaken: These experiments demonstrated that the factors taken in different intermediate and late stages of organogenesis are able to correct the behavior not only of cancer stem cells, but also of the cells involved in other pathological diseases like psoriasis, in which an accelerated multiplication of the keratinocytes is present. Studies in vitro demonstrated that SCDSFs have a very important role in normalizing the growth curve of pathological keratinocytes [40]. Moreover, in clinical trials, a significant amelioration has been demonstrated in the patients treated with phototherapy plus products containing SCDSFs, in comparison with the patients treated with phototherapy alone [41]. Important results were confirmed in other clinical trials on psoriasis [42,43], in which products containing SCDSFs were used.

## 4. The Role of SCDSFs in Addressing the Fate of Human Adipose-Derived Stem Cells (hASCs)

A study of the role of SCDSFs collected from Zebrafish embryos at the early developmental stage, just before the beginning of stem cell differentiation, in determining the fate of human adipose-derived stem cells (hASCs) [44] was conducted. In this research, it was demonstrated that SCDSFs are significant in activating important genes which counteract human cell senescence. Indeed, these factors represent very effective tools to increase stem cell expression of multipotency and to counteract cell senescence, reducing the expression of the beta-galactosidase marker and enhancing the stemness genes *Oct-4*, *Sox-2*, and *c-Myc*. In addition, it was possible to activate the gene expression of TERT, the catalytic subunit of telomerase, and the transcription of Bmi-1 [45], which play a role in counteracting senescence as key telomerase-independent repressors of aging [46,47]. On the basis of the research done on stem cell rejuvenation and differentiation, some studies on the prevention of cell degeneration and on regeneration of tissues without stem cell transplantation were also carried out. Such studies demonstrated that the prevention of cell degeneration is possible only when we administer all the factors present in many different moments of stem cell multiplication and differentiation. This was demonstrated in a recent study on the ability of SCDSFs to prevent neurodegeneration in hippocampal cells of the CA1 area in mice, treated with different toxic stimuli, as already described [48]. In this experiment, it was possible to demonstrate that the prevention of the degeneration of the cells of a tissue is possible when the information is complete and redundant, i.e., only when the information contains all the factors able to regenerate and differentiate the stem cells of a tissue [7]. This and other experiments in which it was demonstrated that an early developmental Zebrafish embryo extract could act as a modulator of senescence in human mesenchymal stem cells (hMSCs) isolated from many adult tissues, as already recorded [8,9,10], could represent the basis for future in vivo approaches promoting rejuvenation and regeneration of different tissues, bypassing stem cell transplantation.

At the clinical level, it has already been possible to confirm these conclusions because in a recent clinical trial of twenty men with androgenetic alopecia treated with SCDSFs containing factors taken from all five stages of cell differentiation, very significant results were obtained. In fact, by means of cryopass-laser treatment for the transdermal administration of SCDSFs, all the treated men demonstrated significant hair follicle regeneration. In a published article [49], the material and methods of the clinical trial, and the role and functioning of the laser to transfer SCDSFs to hair follicles are described. The results were very important: All the patients demonstrated an initial regeneration of hair, in the form of a soft fleece, after the first treatment. This regeneration was consolidated with subsequent treatments and after 10 treatments, the hair took on the consistency of adult, pigmented hair. At a check-up after six months, the number of hairs in the subjects examined was almost unchanged, and there was a general improvement in the number and in the volume of the stems. The treatment did not have any adverse effects and was very well-accepted by the patients, who were satisfied with the results obtained.
